# Supplementary material for: Novel Driver Strength Index highlights important cancer genes in TCGA PanCanAtlas patients
Source: PeerJ. 2022 Aug 11;10:e13860. doi: 10.7717/peerj.13860 (PMC9375969; doi:10.7717/peerj.13860)
Supplement: Supplemental Information 2 [file peerj-10-13860-s002.zip › COHORTS/genes plots/cohorts/2021_8_16_14_9_distribution_cohorts_males_GBM.pdf]

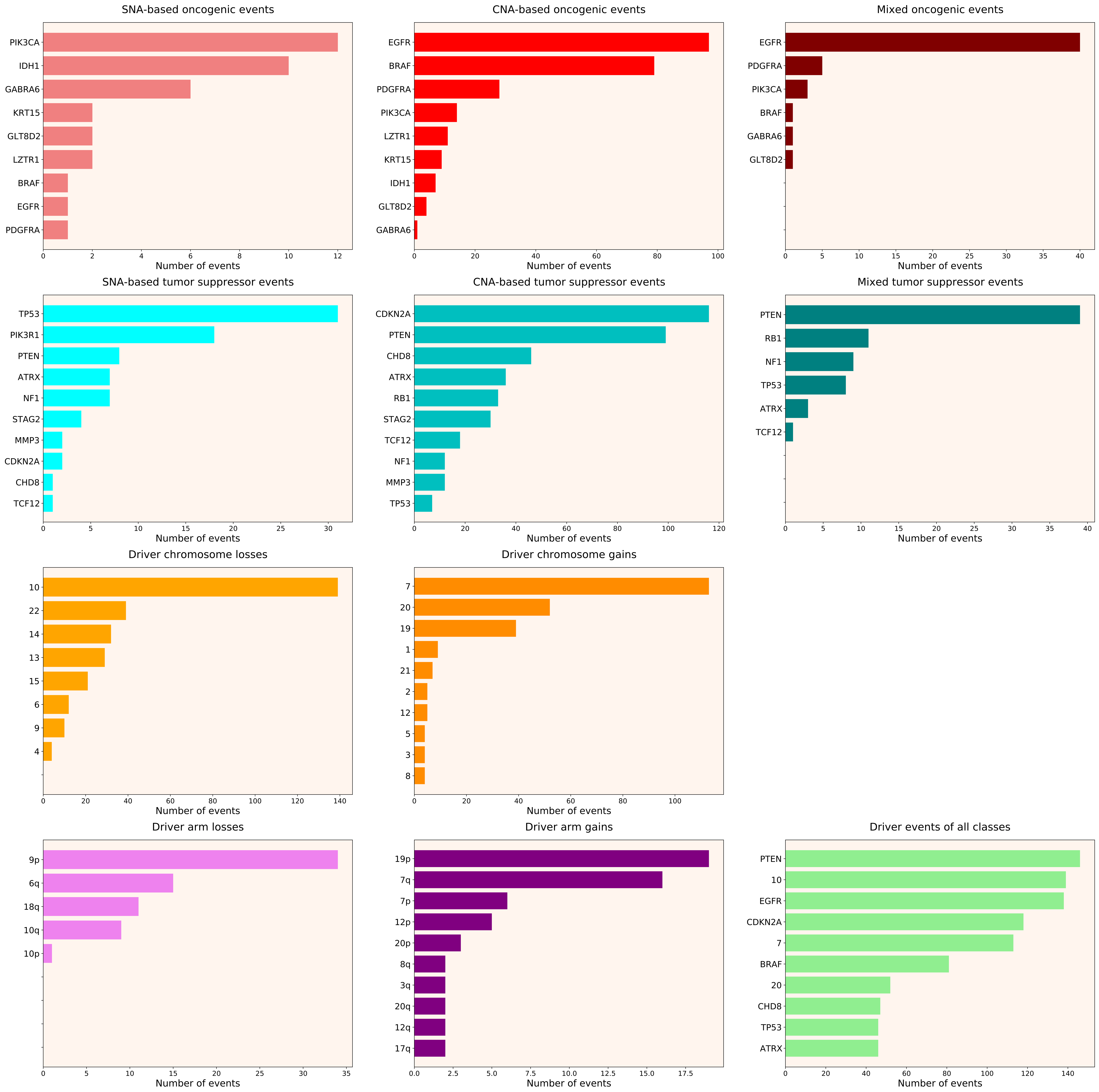
SNA-based oncogenic events
PIK3CA
IDH1
GABRA6
KRT15
GLT8D2
LZTR1
BRAF
EGFR
PDGFRA
0 2 4 6 8 10 12
Number of events
CNA-based oncogenic events
EGFR
BRAF
PDGFRA
PIK3CA
LZTR1
KRT15
IDH1
GLT8D2
GABRA6
0 20 40 60 80 100
Number of events
Mixed oncogenic events
EGFR
PDGFRA
PIK3CA
BRAF
GABRA6
GLT8D2
0 5 10 15 20 25 30 35 40
Number of events
SNA-based tumor suppressor events
TP53
PIK3R1
PTEN
ATRX
NF1
STAG2
MMP3
CDKN2A
CHD8
TCF12
0 5 10 15 20 25 30
Number of events
CNA-based tumor suppressor events
CDKN2A
PTEN
CHD8
ATRX
RB1
STAG2
TCF12
NF1
MMP3
TP53
0 20 40 60 80 100 120
Number of events
Mixed tumor suppressor events
PTEN
RB1
NF1
TP53
ATRX
TCF12
0 5 10 15 20 25 30 35 40
Number of events
Driver chromosome losses
10
22
14
13
15
6
9
4
0 20 40 60 80 100 120 140
Number of events
Driver chromosome gains
7
20
19
1
21
2
12
5
3
8
0 20 40 60 80 100
Number of events
Driver arm losses
9p
6q
18q
10q
10p
0 5 10 15 20 25 30 35
Number of events
Driver arm gains
19p
7q
7p
12p
20p
8q
3q
20q
12q
17q
0.0 2.5 5.0 7.5 10.0 12.5 15.0 17.5
Number of events
Driver events of all classes
PTEN
10
EGFR
CDKN2A
7
BRAF
20
CHD8
TP53
ATRX
0 20 40 60 80 100 120 140
Number of events
